# Phase transitions and self-organized criticality in networks of stochastic spiking neurons

**DOI:** 10.1038/srep35831

**Published:** 2016-11-07

**Authors:** Ludmila Brochini, Ariadne de Andrade Costa, Miguel Abadi, Antônio C. Roque, Jorge Stolfi, Osame Kinouchi

**Affiliations:** 1 Universidade de São Paulo, Departamento de Estatística-IME, São Paulo-SP, 05508-090, Brazil; 2 Universidade de Campinas, Instituto de Computação, Campinas-SP, 13083-852, Brazil; 3 Universidade de São Paulo, Departamento de Física-FFCLRP, Ribeirão Preto-SP, 14040-901, Brazil

## Abstract

Phase transitions and critical behavior are crucial issues both in theoretical and experimental neuroscience. We report analytic and computational results about phase transitions and self-organized criticality (SOC) in networks with general stochastic neurons. The stochastic neuron has a firing probability given by a smooth monotonic function Φ(*V*) of the membrane potential *V*, rather than a sharp firing threshold. We find that such networks can operate in several dynamic regimes (phases) depending on the average synaptic weight and the shape of the firing function Φ. In particular, we encounter both continuous and discontinuous phase transitions to absorbing states. At the continuous transition critical boundary, neuronal avalanches occur whose distributions of size and duration are given by power laws, as observed in biological neural networks. We also propose and test a new mechanism to produce SOC: the use of dynamic neuronal gains – a form of short-term plasticity probably located at the axon initial segment (AIS) – instead of depressing synapses at the dendrites (as previously studied in the literature). The new self-organization mechanism produces a slightly supercritical state, that we called SOSC, in accord to some intuitions of Alan Turing.

“*Another simile would be an atomic pile of less than critical size: an injected idea is to correspond to a neutron entering the pile from without. Each such neutron will cause a certain disturbance which eventually dies away. If, however, the size of the pile is sufficiently increased, the disturbance caused by such an incoming neutron will very likely go on and on increasing until the whole pile is destroyed. Is there a corresponding phenomenon for minds, and is there one for machines? There does seem to be one for the human mind. The majority of them seems to be subcritical, i.e., to correspond in this analogy to piles of subcritical size. An idea presented to such a mind will on average give rise to less than one idea in reply. A smallish proportion are supercritical. An idea presented to such a mind may give rise to a whole “theory” consisting of secondary, tertiary and more remote ideas. (…) Adhering to this analogy we ask, “Can a machine be made to be supercritical?*”” Alan Turing (1950)^1^.

The Critical Brain Hypothesis^2^,^3^ states that (some) biological neuronal networks work near phase transitions because criticality enhances information processing capabilities^4^,^5^,^6^ and health^7^. The first discussion about criticality in the brain, in the sense that subcritical, critical and slightly supercritical branching process of thoughts could describe human and animal minds, has been made in the beautiful speculative 1950 Imitation Game paper by Turing^1^. In 1995, Herz & Hopfield^8^ noticed that self-organized criticality (SOC) models for earthquakes were mathematically equivalent to networks of integrate-and-fire neurons, and speculated that perhaps SOC would occur in the brain. In 2003, in a landmark paper, these theoretical conjectures found experimental support by Beggs & Plenz^9^ and, by now, more than half a thousand papers can be found about the subject, see some reviews^2^,^3^,^10^. Although not consensual, the Critical Brain Hypothesis can be considered at least a very fertile idea.

The open question about neuronal criticality is what are the mechanisms responsible for tuning the network towards the critical state. Up to now, the main mechanism studied is some dynamics in the links which, in the biological context, would occur at the synaptic level^11^,^12^,^13^,^14^,^15^,^16^,^17^.

Here we propose a whole new mechanism: dynamic neuronal gains, related to the diminution (and recovery) of the firing probability, an intrinsic neuronal property. The neuronal gain is experimentally related to the well known phenomenon of firing rate adaptation^18^,^19^,^20^. This new mechanism is sufficient to drive neuronal networks of stochastic neurons towards a critical boundary found, by the first time, for these models. The neuron model we use was proposed by Galves and Locherbach^21^ as a stochastic model of spiking neurons inspired by the traditional integrate-and-fire (IF) model.

Introduced in the early 20th century^22^, IF elements have been extensively used in simulations of spiking neurons^20^,^23^,^24^,^25^,^26^,^27^,^28^. Despite their simplicity, IF models have successfully emulated certain phenomena observed in biological neural networks, such as firing avalanches^12^,^13^,^29^ and multiple dynamical regimes^30^,^31^. In these models, the membrane potential *V*(*t*) integrates synaptic and external currents up to a *firing threshold V*_T_^32^. Then, a spike is generated and *V*(*t*) drops to a *reset potential V*_R_. The *leaky integrate-and-fire* (LIF) model extends the IF neuron with a leakage current, which causes the potential *V*(*t*) to decay exponentially towards a *baseline potential V*_B_ in the absence of input signals^24^,^26^.

LIF models are deterministic but it has been claimed that stochastic models may be more adequate for simulation purposes^33^. Some authors proposed to introduce stochasticity by adding noise terms to the potential^24^,^25^,^30^,^31^,^33^,^34^,^35^,^36^,^37^, yielding the *leaky stochastic integrate-and-fire* (LSIF) models.

Alternatively, the Galves-Löcherbach (GL) model^21^,^38^,^39^,^40^,^41^ and also the model used by Larremore *et al*.^42^,^43^ introduce stochasticity in their firing neuron models in a different way. Instead of noise inputs, they assume that the firing of the neuron is a random event, whose probability of occurrence in any time step is a *firing function* Φ(*V*) of membrane potential *V*. By subsuming all sources of randomness into a single function, the Galves-Löcherbach (GL) neuron model simplifies the analysis and simulation of noisy spiking neural networks.

Brain networks are also known to exhibit *plasticity*: changes in neural parameters over time scales longer than the firing time scale^27^,^44^. For example, short-term synaptic plasticity^45^ has been incorporated in models by assuming that the strength of each synapse is lowered after each firing, and then gradually recovers towards a reference value^12^,^13^. This kind of dynamics drives the synaptic weights of the network towards critical values, a SOC state which is believed to optimize the network information processing^3^,^4^,^7^,^9^,^10^,^46^.

In this work, first we study the dynamics of networks of GL neurons by a very simple and transparent mean-field calculation. We find both continuous and discontinuous phase transitions depending on the average synaptic strength and parameters of the firing function Φ(*V*). To the best of our knowledge, these phase transitions have never been observed in standard integrate-and-fire neurons. We also find that, at the second order phase transition, the stimulated excitation of a single neuron causes avalanches of firing events (neuronal avalanches) that are similar to those observed in biological networks^3^,^9^.

Second, we present a new mechanism for SOC based on a dynamics on the *neuronal gains* (a parameter of the neuron probably related to the axon initial segment – AIS^32^,^47^), instead of depression of coupling strengths (related to neurotransmiter vesicle depletion at synaptic contacts between neurons) proposed in the literature^12^,^13^,^15^,^17^. This new activity dependent gain model is sufficient to achieve self-organized criticality, both by simulation evidence and by mean-field calculations. The great advantage of this new SOC mechanism is that it is much more efficient, since we have only one adaptive parameter per neuron, instead of one per synapse.

## The Model

We assume a network of *N* GL neurons that change states in parallel at certain *sampling times* with a uniform spacing Δ. Thus, the membrane potential of neuron *i* is modeled by a real variable *V*_*i*_[*t*] indexed by *discrete time t*, an integer that represents the sampling time *t*Δ.

Each synapse transmits signals from some *presynaptic* neuron *j* to some *postsynaptic* neuron *i*, and has a *synaptic strength w*_*ij*_. If neuron *j* fires between discrete times *t* and *t* + 1, its potential drops to *V*_R_. This event increments by *w*_*ij*_ the potential of every postsynaptic neuron *i* that does not fire in that interval. The potential of a non-firing neuron may also integrate an *external stimulus I*_*i*_[*t*], which can model signals received from sources outside the network. Apart from these increments, the potential of a non-firing neuron decays at each time step towards the baseline voltage *V*_B_ by a factor *μ* ∈ [0, 1], which models the effect of a leakage current.

We introduce the Boolean variable *X*_*i*_[*t*] ∈ {0, 1} which denotes whether neuron *i* fired between *t* and *t* + 1. The potentials evolve as:


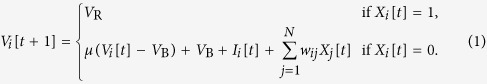


This is a special case of the general GL model^21^, with the filter function 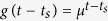, where *t*_*s*_ is the time of the last firing of neuron *i*. We have *X*_*i*_[*t* + 1] = 1 with probability Φ(*V*_*i*_[*t*]), which is called the *firing function*^21^,^38^,^39^,^40^,^41^,^42^. We also have *X*_*i*_[*t* + 1] = 0 if *X*_*i*_[*t*] = 1 (refractory period). The function Φ is sigmoidal, that is, monotonically increasing, with limiting values Φ(−∞) = 0 and Φ(+∞) = 1, with only one derivative maximum. We also assume that Φ(*V*) is zero up to some *threshold potential V*_T_ (possibly −∞) and is 1 starting at some *saturation potential V*_S_ (possibly +∞). If Φ is the shifted Heaviside step function Θ, Φ(*V*) = Θ(*V* − *V*_T_), we have a deterministic discrete-time LIF neuron. Any other choice for Φ(*V*) gives a stochastic neuron.

The network’s activity is measured by the fraction (or density) *ρ*[*t*] of firing neurons:


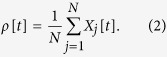


The density *ρ*[*t*] can be computed from the probability density *p*[*t*](*V*) of potentials at time *t*:


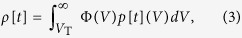


where *p*[*t*](*V*)*dV* is the fraction of neurons with potential in the range [*V*, *V* + *dV*] at time *t*.

Neurons that fire between *t* and *t* + 1 have their potential reset to *V*_R_. They contribute to *p*[*t* + 1](*V*) a Dirac impulse at potential *V*_R_, with amplitude (integral) *ρ*[*t*] given by equation (3). In subsequent time steps, the potentials of all neurons will evolve according to equation (1). This process modifies *p*[*t*](*V*) also for *V* ≠ *V*_R_.

## Results

We will study only fully connected networks, where each neuron receives inputs from all the other *N* − 1 neurons. Since the zero of potential is arbitrary, we assume *V*_B_ = 0. We also consider only the case with *V*_R_ = 0, and uniform constant input *I*_*i*_[*t*] = *I*. So, for these networks, equation (1) reads:


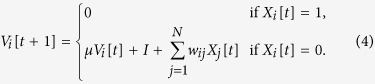


### Mean-field calculation

In the mean-field analysis, we assume that the synaptic weights *w*_*ij*_ follow a distribution with average *W*/*N* and finite variance. The mean-field approximation disregards correlations, so the final term of equation (1) becomes:


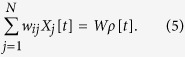


Notice that the variance of the weights *w*_*ij*_ becomes immaterial when *N* tends to infinity.

Since the external input *I* is the same for all neurons and all times, every neuron *i* that does not fire between *t* and *t* + 1 (that is, with *X*_*i*_[*t*] = 0) has its potential changed in the same way:


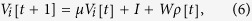


Recall that the probability density *p*[*t*](*V*) has a Dirac impulse at potential *U*_0_ = 0, representing all neurons that fired in the previous interval. This Dirac impulse is modified in later steps by equation (6). It follows that, once all neurons have fired at least once, the density *p*[*t*](*V*) will be a combination of discrete impulses with amplitudes *η*_0_[*t*], *η*_1_[*t*], *η*_2_[*t*], …, at potentials *U*_0_[*t*], *U*_1_[*t*], *U*_2_[*t*], …, such that 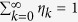.

The amplitude *η*_*k*_[*t*] is the fraction of neurons with *firing age k* at discrete time *t*, that is, neurons that fired between times *t* − *k* − 1 and *t* − *k*, and did not fire between *t* − *k* and *t*. The common potential of those neurons, at time *t*, is *U*_*k*_[*t*]. In particular, *η*_0_[*t*] is the fraction *ρ*[*t* − 1] of neurons that fired in the previous time step. For this type of distribution, the integral of equation (3) becomes a discrete sum:


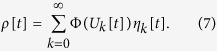


According to equation (6), the values *η*_*k*_[*t*] and *U*_*k*_[*t*] evolve by the equations


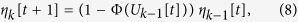



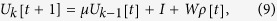


for all *k* ≥ 1, with *η*_0_[*t* + 1] = *ρ*[*t*] and *U*_0_[*t* + 1] = 0.

### Stationary states for general Φ and *μ*

A *stationary state* is a density *p*[*t*](*V*) = *p*(*V*) of membrane potentials that does not change with time. In such a regime, quantities *U*_*k*_ and *η*_*k*_ do not depend anymore on *t*. Therefore, the equations (8) and (9) become the recurrence equations:


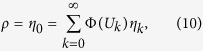



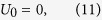



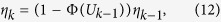



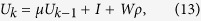


for all *k* ≥ 1.

**Figure 1:**
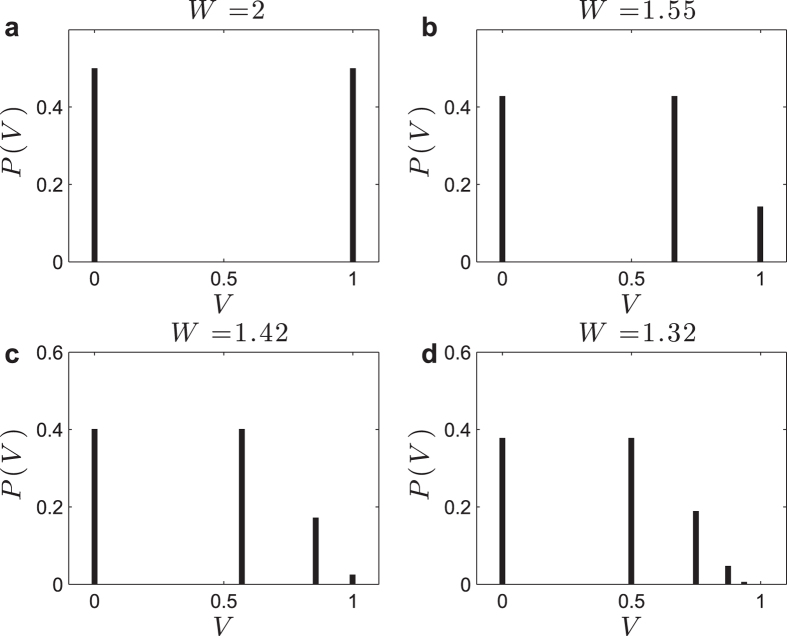
Examples of stationary potential distributions *P*(*V*): monomial Φ function with *r* = 1, Γ = 1, *μ* = 1/2, *I* = 0 case with different values of *W*. (**a**) *W*_2_ = *W*_B_ = 2, two peaks; (**b**) *W*_3_ = 14/9, three peaks; (**c**) *W*_4_ = 488/343, four peaks, (**d**) *W*_∞_ ≈ 1.32, infinite number of peaks with *U*_∞_ = 1. Notice that for *W* < *W*_∞_ all the peaks in the distribution *P*(*V*) lie at potentials *U*_*k*_ < 1. For *W*_B_ = 2 we have *η*_0_ = *η*_1_ = 1/2, producing a bifurcation to a *2-cycle*. The values of *W*_*m*_ = *W*_2_, *W*_3_, *W*_4_ and *W*_∞_ can be obtained analytically by imposing the condition *U*_*m*__−1_ = 1 in equations (12–13).

Since equations (12) are homogeneous on the *η*_*k*_, the normalization condition 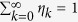 must be included explicitly. So, integrating over the density *p*(*V*) leads to a discrete distribution *P*(*V*) (see Fig. 1 for a specific Φ).

Equations (10, 11, 12, 13) can be solved numerically, e. g. by simulating the evolution of the potential probability density *p*[*t*](*V*) according to equations (8) and (9), starting from an arbitrary initial distribution, until reaching a stable distribution (the probabilities *η*_*k*_ should be renormalized for unit sum after each time step, to compensate for rounding errors). Notice that this can be done for any Φ function, so this numerical solution is very general.

### The monomial saturating Φ with *μ* > 0

Now we consider a specific class of firing functions, the *saturating monomials*. This class is parametrized by a positive *degree r* and a *neuronal gain* Γ > 0. In all functions of this class, Φ(*V*) is 0 when *V* ≤ *V*_T_, and 1 when *V* ≥ *V*_S_, where the saturation potential is *V*_S_ = *V*_T_ + 1/Γ. In the interval *V*_T_ < *V* < *V*_S_, we have:


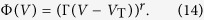


Note that these functions can be seen as limiting cases of sigmoidal functions, and that we recover the deterministic LIF model Φ(*V*) = Θ(*V* − *V*_T_) when Γ → ∞.

For any integer *p* ≥ 2, there are combinations of values of *V*_T_, *V*_S_, and *μ* that cause the network to behave deterministically. This happens if the stationary state defined by equations (12) and (13) is such that *U*_*p*−2_ ≤ *V*_T_ ≤ *V*_S_ ≤ *U*_*p*−1_—that is, Φ(*U*_*k*_) is either 0 or 1 for all *k*, so the GL model becomes equivalent to the deterministic LIF model. In such a stationary state, we have *ρ* = *η*_*k*_ = 1/*p* for all *k* < *p*; meaning that the neurons are divided into *p* groups of equal size, and each group fires every *p* steps, exactly. If the inequalities are strict (*U*_*p*−2_ < *V*_T_ and *V*_S_ < *U*_*p*−1_) then there are also many deterministic periodic regimes (*p-cycles*) where the *p* groups have slightly more or less than 1/*p* of all the neurons, but still fire regularly every *p* steps.

Note that, if *V*_T_ = 0, such degenerate (deterministic) regimes, stationary or periodic, occur only for *p* = 2 and *W* ≥ *W*_B_ where *W*_B_ = 2(*I* + *V*_S_). The stationary regime has *ρ* = *η*_0_ = *η*_1_ = 1/2 and *U*_1_ = *I* + *W*/2. In the periodic regimes (*2-cycles*) the activity *ρ*[*t*] alternates between two values *ρ*′ and *ρ*′′ = 1 − *ρ*′, with *ρ*_1_(*W*) < *ρ*′ < 1/2 < *ρ*′′ < *ρ*_2_(*W*), where:


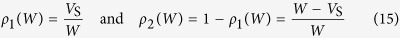


All these *2-cycles* are marginally stable, in the sense that, if a perturbed state *ρ*_*ε*_ = *ρ* + *ε* satisfy equation (15) then the new cycle *ρ*_*ε*_[*t* + 1] = 1 − *ρ*_*ε*_[*t*] is also marginally stable.

**Figure 2:**
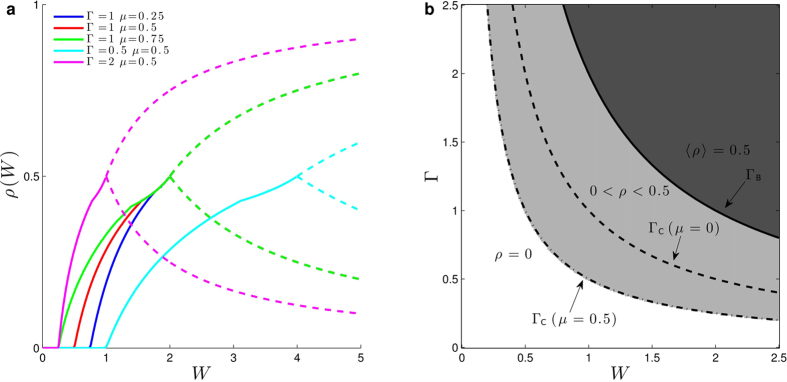
Results for *μ* > 0. (**a**) Numerically computed *ρ*(*W*) curves for the monomial Φ with *r* = 1, *I* = *V*_R_ = *V*_T_ = 0, and (Γ, *μ*) = (1, 1/4), (1, 1/2), (1, 3/4), (1/2, 1/2), and (2, 1/2). The absorbing state *ρ*_0_ = 0 looses stability at *W*_C_ and the non trivial fixed point *ρ* > 0 appears. At *W*_B_ = 2/Γ, we have *ρ* = 1/2 and from there we have the fixed point *ρ*[*t*] = 1/2 and the *2-cycles* with *ρ*[*t*] between the two bounds of equation (15) (dashed lines). (**b**) Numerically computed (Γ, *W*) diagram showing the critical boundaries Γ_C_(*W*) = (1 − *μ*)/*W* and the bifurcation line Γ_B_(*W*) = 2/*W* to *2-cycles*.

In the analyses that follows, the control parameters are *W* and Γ, and *ρ*(*W*, Γ) is the order parameter. We obtain numerically *ρ*(*W*, Γ) and the phase diagram (*W*, Γ) for several values of *μ* > 0, for the linear (*r* = 1) saturating Φ with *I* = *V*_T_ = 0 (Fig. 2). Only the first 100 peaks (*U*_*k*_, *η*_*k*_) were considered, since, for the given *μ* and Φ, there was no significant probability density beyond that point. The same numerical method can be used for *r* ≠ 1, *I* ≠ 0, *V*_T_ ≠ 0.

Near the critical point, we obtain numerically *ρ*(*W*, *μ*) ≈ *C*(*W* − *W*_C_)/*W*, where *W*_C_(Γ) = (1 − *μ*)/Γ and *C*(*μ*) is a constant. So, the critical exponent is *α* = 1, characteristic of the mean-field directed percolation (DP) universality class^3^,^4^. The critical boundary in the (*W*, Γ) plane, numerically obtained, seems to be Γ_C_(*W*) = (1 − *μ*)/*W* (Fig. 2b).

### Analytic results for *μ* = 0

Below we give results of a simple mean-field analysis in the limits *N* → ∞ and *μ* → 0. The latter implies that, at time *t* + 1, the neuron “forgets” its previous potential *V*_*i*_[*t*] and integrates only the inputs *I*[*t*] + *W*_*ij*_*X*_*j*_[*t*]. This scenario is interesting because it enables analytic solutions, yet exhibits all kinds of behaviors and phase transitions that occur with *μ* > 0.

When *μ* = 0 and *I*_*i*_[*t*] = *I* (uniform constant input), the density *p*[*t*](*V*) consists of only two Dirac peaks at potentials *U*_0_[*t*] = *V*_R_ = 0 and *U*_1_[*t*] = *I* + *Wρ*[*t* − 1], with fractions *η*_0_[*t*] and *η*_1_[*t*] that evolve as:


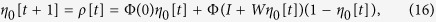



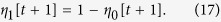


Furthermore, if the neurons cannot fire spontaneously, that is, Φ(0) = 0, then equation (16) reduces to:


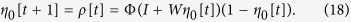


In a stationary regime, equation (18) simplifies to:


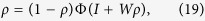


since *η*_0_ = *ρ*, *η*_1_ = 1 − *ρ*, *U*_0_ = 0, and *U*_1_ = *I* + *Wρ*. Below, all the results refer to the monomial saturating Φs given by equation (14).

### The case with *r* = 1, *V*_T_ = 0

**Figure 3:**
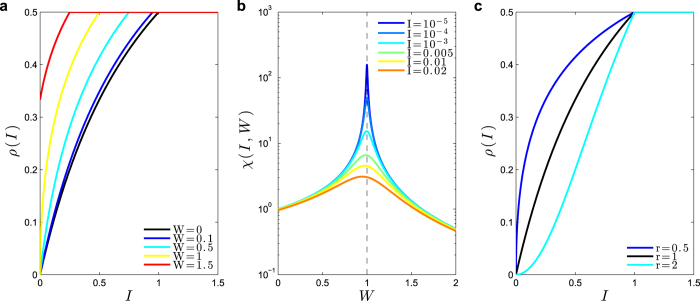
Network and isolated neuron responses to external input *I*. (**a**) Network activity *ρ*(*I*, *W*) as a function of *I* for several *W*; (**b**) Susceptibility *χ*(*I*, *W*) as a function of *W* for several *I*. Notice the divergence *χ*_C_(*I*) ∝ *I*^−1/2^ for small *I*; (**c**) Firing rate of an isolated neuron *ρ*(*I*, *W* = 0) for monomial exponents *r* = 0.5, 1 and 2.

When *r* = 1, we have the linear function Φ(*V*) = Γ*V* for 0 < *V* < *V*_S_ = 1/Γ, where *V* = *I* + *Wρ*. Equation (19) turns out:


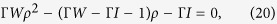


with solution (Fig. 3a):


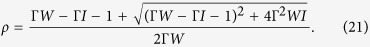


For zero input we have:


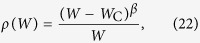


where *W*_C_ = 1/Γ and the order parameter critical exponent is *β* = 1. This corresponds to a standard mean-field continuous (second order) absorbing state phase transition. This transition will be studied in detail two section below.

A measure of the network sensitivity to inputs (which play here the role of external fields) is the susceptibility *χ* = *dρ*/*dI*, which is a function of Γ, *W* and *I* (Fig. 3b):


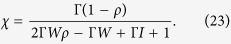


For zero external inputs, the susceptibility behaves as:


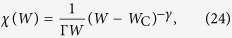


where we have the critical exponent *γ* = 1.

A very interesting result is that, for any *I*, the susceptibility is maximized at the critical line *W*_C_ = 1/Γ, with the values:


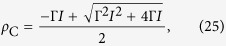



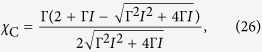


For *I* → 0 we have 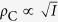. The critical exponent *δ* is defined by *I* ∝ *ρ*^*δ*^ for small *I*, so we obtain the mean-field value *δ* = 2. In analogy with Psychophysics, we may call *m* = 1/*δ* = 1/2 the Stevens’s exponent of the network^4^.

With two critical exponents it is possible to obtain others through scaling relations. For example, notice that *β*, *γ* and *δ* are related to 2*β* + *γ* = *β*(*δ* + 1).

Notice that, at the critical line, the susceptibility diverges as 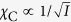 as *I* → 0. We will comment the importance of the fractionary Stevens’s exponent *m* = 1/2 (Fig. 3a) and the diverging susceptibility (Fig. 3b) for information processing in the Discussion section.

#### Isolated neurons

We can also analyze the behavior of the GL neuron model under the standard experiment where an isolated neuron *in vitro* is artificially injected with a current of constant intensity *J*. That corresponds to setting the external input signal *I*[*t*] of that neuron to a constant value *I* = *J*Δ/*C* where *C* is the effective capacitance of the neuron.

The firing rate of an isolated neuron can be written as:


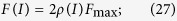


where *F*_max_ is an empirical maximum firing rate (measured in spikes per second) of a given neuron and *ρ* is our previous neuron firing probability per time step. With *W* = 0 and *I* > 0 in equation (19), we get:


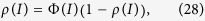


The solution for the monomial saturating Φ with *V*_T_ = 0 is (Fig. 3c):


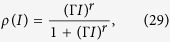


which is less than *ρ* = 1/2 only if *I* < 1/Γ. For any *I* ≥ 1/Γ the firing rate saturates at *ρ* = 1/2 (the neuron fires at every other step, alternating between potentials *U*_0_ = *V*_R_ = 0 and *U*_1_ = *I*. So, for *I* > 0, there is no phase transition. Interestingly, equation (29), known as generalized Michaelis-Menten function, is frequently used to fit the firing response of biological neurons to DC currents^48^,^49^.

#### Continuous phase transitions in networks: the case with *r* = 1

Even with *I* = 0, spontaneous collective activity is possible if the network suffers a phase transition. With *r* = 1, the stationary state condition equation (19) is:


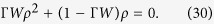


**Figure 4:**
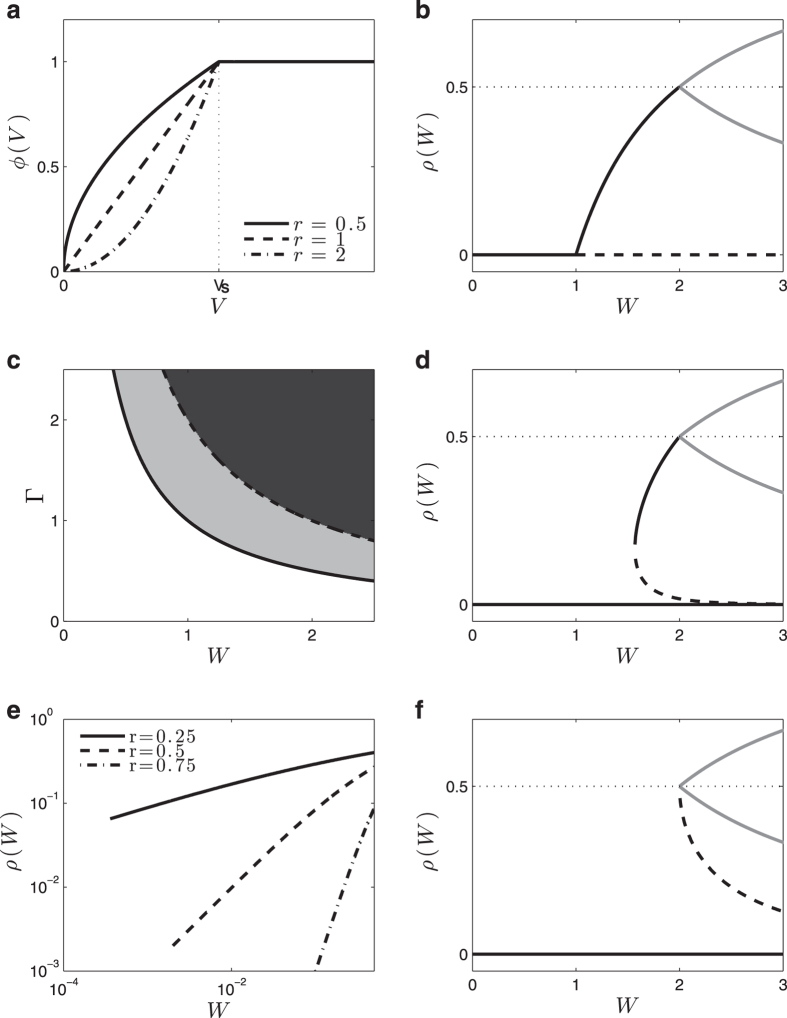
Firing densities (with Γ = 1) and phase diagram with *μ* = 0 and V_T_ (*I*) = 0. (**a**) Examples of monomial firing functions Φ(*V*) with Γ = 1 *r* = 0.5, 1 and 2. (**b**) The *ρ*(*W*) bifurcation plot for *r* = 1. The absorbing state *ρ*_0_ looses stability after *W* > *W*_C_ = 1 (dashed line). The non trivial fixed point *ρ* bifurcates at *W*_B_ = 2/Γ = 2 into two branches (gray lines) that bound the marginally stable *2-cycles*. (**c**) The (Γ, *W*) phase diagram for *r* = 1. Below the critical boundary Γ = Γ_C_(*W*) = 1/*W* the inactive state *ρ*_0_ = 0 is absorbing and stable; above that line it is also absorbing but unstable. Above the line Γ = Γ_B_(*W*) = 2/*W* there are only the marginally stable *2-cycles*. For Γ_C_(*W*) < Γ ≤ Γ_B_(*W*) there is a single stationary regime *ρ*(*W*) = (*W* − *W*_C_)/*W* < 1/2, with *W*_C_ = 1/Γ. (**d**) Discontinuous phase transitions for Γ = 1 with exponents *r* = 1.2. The absorbing state *ρ*_0_ now is stable (solid line at zero). The non trivial fixed point *ρ*^+^ starts with the value *ρ*_C_ at *W*_C_ and bifurcates at *W*_B_, creating the boundary curves (gray) that delimit possible *2-cycles*. At *W*_C_ also appears the unstable separatrix *ρ*_−_ (dashed line). (**e**) Ceaseless activity (no phase transitions) for *r* = 0.25, 0.5 and *r* = 0.75. The activity approach zero (for *W* = 0) as power laws. (**f**) In the limiting case *r* = 2 we do not have a *ρ* > 0 fixed point, but only the stable *ρ*_0_ = 0 (black), the *2-cycles* region (gray) and the unstable separatrix (traces).

The two solutions are the absorbing state *ρ*_0_ = 0 and the non-trivial state:


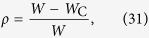


with *W*_C_ = 1/Γ. Since we must have 0 < *ρ* ≤ 1/2, this solution is valid only for *W*_C_ < *W* ≤ *W*_B_ = 2/Γ (Fig. 4b).

This solution describes a stationary state where 1 − *ρ* of the neurons are at potential *U*_1_ = *W* − *W*_C_. The neurons that will fire in the next step are a fraction Φ(*U*_1_) of those, which are again a fraction *ρ* of the total. For any *W* > *W*_C_, the state *ρ*_0_ = 0 is unstable: any small perturbation of the potentials cause the network to converge to the active stationary state above. For *W* < *W*_C_, the solution *ρ*_0_ = 0 is stable and absorbing. In the *ρ*(*W*) plot, the locus of stationary regimes defined by equation (31) bifurcates at *W* = *W*_B_ into the two bounds of equation (15) that delimit the *2-cycles* (Fig. 4b).

So, at the critical boundary *W* = 1/Γ, we have a standard continuous absorbing state transition *ρ*(*W*) ∝ (*W* − *W*_C_)^*α*^ with a critical exponent *α* = 1, which also can be written as *ρ*(Γ) ∝ (Γ − Γ_C_)^*α*^. In the (Γ, *W*) plane, the phase transition corresponds to a critical boundary Γ_C_(*W*) = 1/*W*, below the *2-cycle* phase transition Γ_B_(*W*) = 2/*W* (Fig. 4c).

#### Discontinuous phase transitions in networks: the case with *r* > 1

When *r* > 1 and *W* ≤ *W*_B_ = 2/Γ, the stationary state condition is:


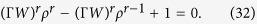


This equation has a non trivial solution *ρ*^+^ only when 1 ≤ *r* ≤ 2 and *W*_C_(*r*) ≤ *W* ≤ *W*_B_, for a certain *W*_C_(*r*) > 1/Γ. In this case, at *W* = *W*_C_(*r*), there is a discontinuous (first-order) phase transition to a regime with activity *ρ* = *ρ*_C_(*r*) ≤ 1/2 (Fig. 4d). It turns out that *ρ*_C_(*r*) → 0 as *r* → 1, recovering the continuous phase transition in that limit. For *r* = 2, the solution to equation (32) is a single point *ρ*(*W*_C_) = *ρ*_C_ = 1/2 at *W*_C_ = 2/Γ = *W*_B_ (Fig. 4f).

Notice that, in the linear case, the fixed point *ρ*_0_ = 0 is unstable for *W* > 1 (Fig. 4b). This occurs because the *separatrix ρ*_−_ (trace lines, Fig. 4d), for *r* → 1, collapses with the *ρ*_0_ point, so that it looses its stability.

#### Ceaseless activity: the case with *r* < 1

When *r* < 1, there is no absorbing solution *ρ*_0_ = 0 to equation (32). In the *W* → 0 limit we get *ρ*(*W*) = (Γ*W*)^*r*/(1−*r*)^. These power laws means that *ρ* > 0 for any *W* > *W*_C_(*r*) = 0 (Fig. 4e). We recover the second order transition *W*_C_(*r* = 1) = 1/Γ when *r* → 1 in equation (32). Interestingly, this ceaseless activity *ρ* > 0 for any *W* > 0 seems to be similar to that found by Larremore *et al*.^42^ with a *μ* = 0 linear saturating model. That ceaseless activity, observed even with *r* = 1, perhaps is due to the presence of inhibitory neurons in Larremore *et al*. model.

#### Discontinuous phase transitions in networks: the case with *V*_T_ > 0 and *I* > 0

The standard IF model has *V*_T_ > 0. If we allow this feature in our models we find a new ingredient that produces first order phase transitions. Indeed, in this case, if *U*_1_ = *Wρ* + *I* < *V*_T_ then we have a single peak at *U*_0_ = 0 with *η*_0_ = 1, which means we have a silent state. When *U*_1_ = *Wρ* + *I* > *V*_T_, we have a peak with height *η*_1_ = 1 − *ρ* and *ρ* = *η*_0_ = Φ(*U*_1_)*η*_1_.

**Figure 5:**
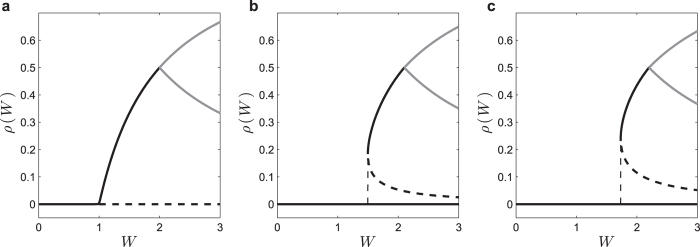
Phase transitions for *V*_T_ > 0: monomial model with *μ* = 0, *r* = 1, Γ = 1 and thresholds *V*_T_ = 0, 0.05 and 0.1. Here the solid black lines represent the stable fixed points, dashed black lines represent unstable fixed points and grey lines correspond to the marginally stable boundaries for *2-cycles* regime. The discontinuity *ρ*_C_ goes to zero for *V*_T_ → 0.

For the linear monomial model this leads to the equations:


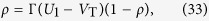



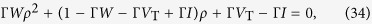


with the solution:


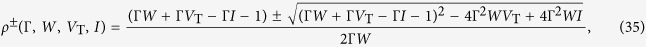


where *ρ*^+^ is the non trivial fixed point and *ρ*^−^ is the unstable fixed point (separatrix). These solutions only exist for Γ*W* values such that 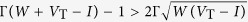. This produces the condition:


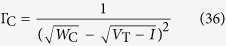


which defines a first order critical boundary. At the critical boundary the density of firing neurons is:


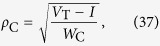


which is nonzero (discontinuous) for any *V*_T_ > *I*. These transitions can be seen in Fig. 5. The solutions for equations (35) and (37) is valid only for *ρ*_C_ < 1/2 (*2-cycle* bifurcation). This imply the maximal value *V*_T_ = *W*_C_ /4 + *I*.

### Neuronal avalanches

**Figure 6:**
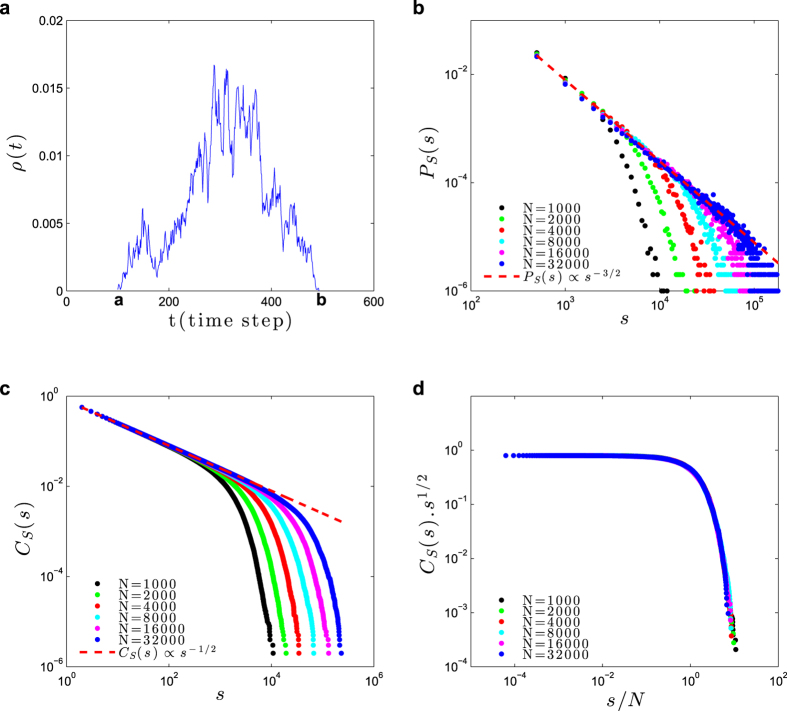
Avalanche size statistics in the static model. Simulations at the critical point *W*_C_ = 1, Γ_C_ = 1 (with *μ* = 0). (**a**) Example of avalanche profile *ρ*[*t*] at the critical point. (**b**) Avalanche size distribution *P*_*S*_(*s*) ≡ *P*(*S* = *s*), for network sizes *N* = 1000, 2000, 4000, 8000, 16000 and 32000. The dashed reference line is proportional to 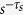, with *τ*_*s*_ = 3/2. (**c**) Complementary cumulative distribution 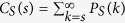. Being an integral of *P*_*S*_(*s*), its power law exponent is −*τ*_*s*_ + 1 = −1/2 (dashed line). (**d**) Data collapse (finite-size scaling) for *C*_*S*_(*s*)*s*^1/2^ versus function of 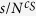, with the cutoff exponent *c*_*S*_ = 1.

Firing avalanches in neural networks have attracted significant interest because of their possible connection to efficient information processing^3^,^4^,^5^,^7^,^9^. Through simulations, we studied the critical point *W*_C_ = 1, Γ_C_ = 1 (with *μ* = 0) in search for neuronal avalanches^3^,^9^ (Fig. 6).

An avalanche that starts at discrete time *t* = *a* and ends at *t* = *b* has duration *d* = *b* − *a* and size 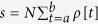 (Fig. 6a). By using the notation *S* for a random variable and *s* for its numerical value, we observe a power law avalanche size distribution 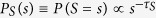, with the mean-field exponent *τ*_*S*_ = 3/2 (Fig. 6b)^3^,^9^,^13^. Since the distribution *P*_*S*_(*s*) is noisy for large *s*, for further analysis we use the complementary cumulative function 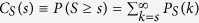 (which gives the probability of having an avalanche with size equal or greater than *s*) because it is very smooth and monotonic (Fig. 6c). Data collapse gives a finite-size scaling exponent *c*_*S*_ = 1 (Fig. 6d)^15^,^17^.

**Figure 7:**
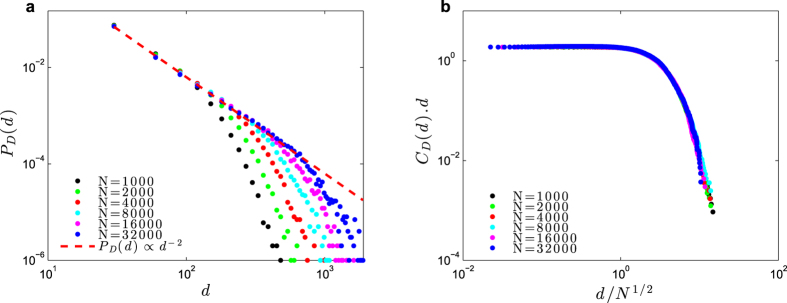
Avalanche duration statistics in the static model. Simulations at the critical point *W*_C_ = 1, Γ_C_ = 1 (*μ* = 0) for network sizes *N* = 1000, 2000, 4000, 8000, 16000 and 32000: (**a**) Probability distribution *P*_*D*_(*d*) ≡ *P*(*D* = *d*) for avalanche duration *d*. The dashed reference line is proportional to 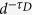, with *τ*_*D*_ = 2. (**b**) Data collapse *C*_*D*_(*d*)*d* versus 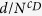, with the cutoff exponent *c*_*D*_ = 1/2. The complementary cumulative function 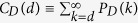, being an integral of *P*_*D*_(*d*), has power law exponent −*τ*_*D*_ + 1 = −1.

We also observed a power law distribution for avalanche duration, 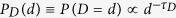 with *τ*_*D*_ = 2 (Fig. 7a). The complementary cumulative distribution is 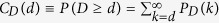. From data collapse, we find a finite-size scaling exponent *c*_*D*_ = 1/2 (Fig. 7b), in accord with the literature^13^.

### The model with dynamic parameters

The results of the previous section were obtained by fine-tuning the network at the critical point Γ_C_ = *W*_C_ = 1. Given the conjecture that the critical region presents functional advantages, a biological model should include some homeostatic mechanism capable of tuning the network towards criticality. Without such mechanism, we cannot truly say that the network *self-organizes* toward the critical regime.

However, observing that the relevant parameter for criticality in our model is the critical boundary Γ_C_*W*_C_ = 1, we propose to work with dynamic gains Γ_*i*_[*t*] while keeping the synapses *W*_*ij*_ fixed. The idea is to reduce the gain Γ_*i*_[*t*] when the neuron fires, and let the gain slowly recover towards a higher resting value after that:


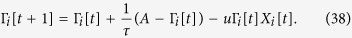


Now, the factor *τ* is related to the characteristic recovery time of the gain, *A* is the asymptotic resting gain, and *u* ∈ [0, 1] is the fraction of gain lost due to the firing. This model is plausible biologically, and can be related to a decrease and recovery, due to the neuron activity, of the firing probability at the AIS^47^. Our dynamic Γ_*i*_[*t*] mimics the well known phenomenon of *spike frequency adaptation*^18^,^19^.

**Figure 8:**
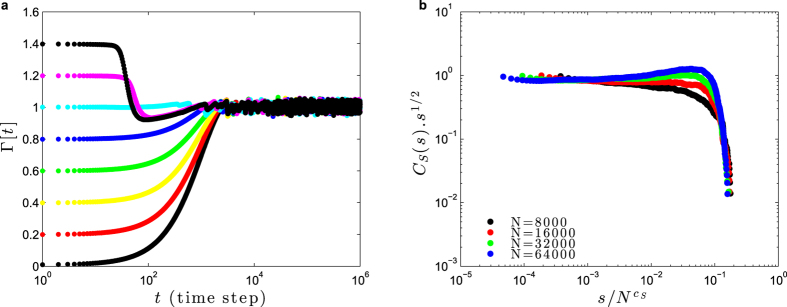
Self-organization with dynamic neuronal gains. Simulations of a network of GL neurons with fixed *W*_*ij*_ = *W* = 1, *u* = 1, *A* = 1.1 and *τ* = 1000 ms. Dynamic gains Γ_*i*_[*t*] starts with Γ_*i*_[0] uniformly distributed in [0, Γ_max_]. The average initial condition is 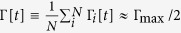, which produces the different initial conditions Γ[0]. (**a**) Self-organization of the average gain Γ[*t*] over time. The horizontal dashed line marks the value Γ_C_ = 1. (**b**) Data collapse for *C*_*S*_(*s*)*s*^1/2^ versus 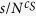 for several *N*, with the cutoff exponent *c*_*S*_ = 1.

Figure 8a shows a simulation with all-to-all coupled networks with *N* neurons and, for simplicity, *W*_*ij*_ = *W*. We observe that the average gain 
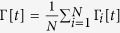
 seems to converge toward the critical value Γ_C_(*W*) = 1/*W* = 1, starting from different Γ[0] ≠ 1. As the network converges to the critical region, we observe power-law avalanche size distributions with exponent −3/2 leading to a cumulative function *C*_*S*_(*s*) ∝ *s*^−1/2^ (Fig. 8b). However, we also observe supercritical bumps for large *s* and *N*, meaning that the network is in a slightly supercritical state.

This empirical evidence is supported by a mean-field analysis of equation (38). Averaging over the sites, we have for the average gain:


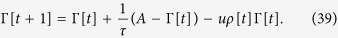


In the stationary state, we have Γ[*t* + 1] = Γ[*t*] = Γ^*^, so:


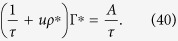


But we have the relation


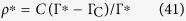


near the critical region, where *C* is a constant that depends on Φ(*V*) and *μ*, for example, with *μ* = 0, *C* = 1 for Φ linear monomial model. So:


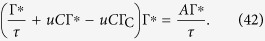


Eliminating the common factor Γ^*^, and dividing by *uC*, we have:


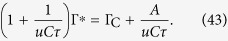


Now, call *x* = 1/(*uCτ*). Then, we have:


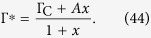


The fine tuning solution is to put by hand *A* = Γ_C_, which leads to Γ^*^ = Γ_C_ independent of *x*. This fine tuning solution should not be allowed in a true SOC scenario. So, suppose that *A* = *B*Γ_C_. Then, we have:


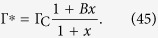


Now we see that to have a critical or supercritical state (where equation (41) holds) we must have *B* > 1, otherwise we fall in the subcritical state Γ^*^ < Γ_C_ where ρ^*^ = 0 and our mean-field calculation is not valid. A first order approximation leads to:


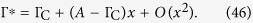


This mean-field calculation shows that, if *x* → 0, we obtain a SOC state Γ^*^ → Γ_C_. However, the strict case *x* → 0 would require a scaling *τ* = *O*(*N*^*a*^) with an exponent *a* > 0, as done previously for dynamic synapses^12^,^13^,^15^,^17^.

However, if we want to avoid the non-biological scaling *τ*(*N*) = *O*(*N*^*a*^), we can use biologically reasonable parameters like *τ* ∈ [10, 1000] ms, *u* = [0.1, 1], *C* = 1 and *A* ∈ [1.1, 2]Γ_C_. In particular, if *τ* = 1000, *u* = 1 and *A* = 1.1, we have *x* = 0.001 and:


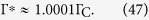


Even a more conservative value *τ* = 100 ms gives Γ^*^ ≈ 1.001Γ_C_. Although not perfect SOC^10^, this result is totally sufficient to explain power law neuronal avalanches. We call this phenomena self-organized supercriticality (SOSC), where the supercriticality can be very small. We must yet determine the volume of parameter space (*τ*, *A*, *u*) where the SOSC phenomenon holds. In the case of dynamic synapses *W*_*ij*_[*t*], this parametric volume is very large^15^,^17^ and we conjecture that the same occurs for the dynamic gains Γ_*i*_[*t*]. This shall be studied in detail in another paper.

## Discussion

### Stochastic model

The stochastic neuron Galves and Löcherbach^21^,^41^ is an interesting element for studies of networks of spiking neurons because it enables exact analytic results and simple numerical calculations. While the LSIF models of Soula *et al*.^34^ and Cessac^35^,^36^,^37^ introduce stochasticity in the neuron’s behavior by adding noise terms to its potential, the GL model is agnostic about the origin of noise and randomness (which can be a good thing when several noise sources are present). All the random behavior is grouped at the single firing function Φ(*V*).

### Phase transitions

Networks of GL neurons display a variety of dynamical states with interesting phase transitions. We looked for stationary regimes in such networks, for some specific firing functions Φ(*V*) with no spontaneous activity at the baseline potential (that is, with Φ(0) = 0 and *I* = 0). We studied the changes in those regimes as a function of the mean synaptic weight *W* and mean neuronal gain Γ. We found basically tree kinds of phase transition, depending of the behavior of Φ(*V*) ∝ *V*^*r*^ for low *V*:

*r* < 1: A ceaseless dynamic regime with no phase transitions (*W*_C_ = 0) similar to that found by Larremore *et al*.^42^;

*r* = 1: A continuous (second order) absorbing state phase transition in the Directed Percolation universality class usual in SOC models^2^,^3^,^10^,^15^,^17^;

*r* > 1: Discontinuous (first order) absorbing state transitions.

We also observed discontinuous phase transitions for any *r* > 0 when the neurons have a firing threshold *V*_T_ > 0.

The deterministic LIF neuron models, which do not have noise, do not seem to allow these kinds of transitions^27^,^30^,^31^. The model studied by Larremore *et al*.^42^ is equivalent to the GL model with monomial saturating firing function with *r* = 1, *V*_T_ = 0, *μ* = 0 and Γ = 1. They did not report any phase transition (perhaps because of the effect of inhibitory neurons in their network), but found a ceaseless activity very similar to what we observed with *r* < 1.

### Avalanches

In the case of second-order phase transitions (Φ(0) = 0, *r* = 1, *V*_T_ = 0), we detected firing avalanches at the critical boundary Γ_C_ = 1/*W* whose size and duration power law distributions present the standard mean-field exponents *τ*_*S*_ = 3/2 and *τ*_*D*_ = 2. We observed a very good finite-scaling and data collapse behavior, with finite-size exponents *c*_*S*_ = 1 and *c*_*D*_ = 1/2.

### Maximal susceptibility and optimal dynamic range at criticality

Maximal susceptibility means maximal sensitivity to inputs, in special to weak inputs, which seems to be an interesting property in biological terms. So, this is a new example of optimization of information processing at criticality. We also observed, for small *I*, the behavior *ρ*(*I*) ∝ *I*^*m*^ with a fractionary Stevens’s exponent *m* = 1/*δ* = 1/2. Fractionary Stevens’s exponents maximize the network dynamic range since, outside criticality, we have only a input-output proportional behavior *ρ*(*I*) ∝ *I*, see ref. 4. As an example, in non-critical systems, an input range of 1–10000 spikes/s, arriving to the neurons due to their extensive dendritic arbors, must be mapped onto a range also of 1–10000 spikes/s in each neuron, which is biologically impossible because neuronal firing do not span four orders of magnitude. However, at criticality, since 
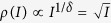
, a similar input range needs to be mapped only to an output range of 1–100 spikes/s, which is biologically possible. Optimal dynamic range and maximal susceptibility to small inputs constitute prime biological motivations to neuronal networks self-organize toward criticality.

### Self-organized criticality

One way to achieve this goal is to use dynamical synapses *W*_*ij*_[*t*], in a way that mimics the loss of strength after a synaptic discharge (presumably due to neurotransmitter vesicles depletion), and the subsequent slow recovery^12^,^13^,^15^,^17^:


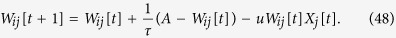


The parameters are the synaptic recovery time *τ*, the asymptotic value *A*, and the fraction *u* of synaptic weight lost after firing. This synaptic dynamics has been examined in refs 12, 13, 15 and 17. For our all-to-all coupled network, we have *N*(*N* − 1) dynamic equations for the *W*_*ij*_*s*. This is a huge number, for example *O*(10^8^) equations, even for a moderate network of *N* = 10^4^ neurons^15^,^17^. The possibility of well behaved SOC in bulk dissipative systems with loading is discussed in refs 10, 13 and 50. Further considerations for systems with conservation on the average at the stationary state, as occurs in our model, are made in refs 15 and 17.

Inspired by the presence of the critical boundary, we proposed a new mechanism for short-scale neural network plasticity, based on dynamic neuron gains Γ_*i*_[*t*] instead of the above dynamic synaptic weights. This new mechanism is biologically plausible, probably related an activity-dependent firing probability at the axon initial segment (AIS)^32^,^47^, and was found to be sufficient to self-organize the network near the critical region. We obtained good data collapse and finite-size behavior for the *P*_*S*_(*S*) distributions.

The great advantage of this new SOC mechanism is its computational efficiency: when simulating *N* neurons with *K* synapses each, there are only *N* dynamic equations for the gains Γ_*i*_[*t*], instead of *NK* equations for the synaptic weights *W*_*ij*_[*t*]. Notice that, for the all-to-all coupling network studied here, this means *O*(*N*^2^) equations for dynamic synapse but only *O*(*N*) equations for dynamic gains. This makes a huge difference for the network sizes that can be simulated.

We stress that, since we used *τ* finite, the criticality is not perfect (Γ^*^/Γ_C_ ∈ [1.001; 1.01]). So, we called it a self-organized super-criticality (SOSC) phenomenon. Interestingly, SOSC would be a concretization of Turing’s intuition that the best brain operating point is slightly supercritical^1^.

We speculate that this slightly supercriticality could explain why humans are so prone to supercritical pathological states like epilepsy^3^ (prevalence 1.7%) and mania (prevalence 2.6% in the population). Our mechanism suggests that such pathological states arises from small gain depression *u* or small gain recovery time *τ*. These parameters are experimentally related to firing rate adaptation and perhaps our proposal could be experimentally studied in normal and pathological tissues.

We also conjecture that this supecriticality in the whole network could explain the *Subsamplig Paradox* in neuronal avalanches: since the initial experimental protocols^9^,^10^, critical power laws have been seem when using arrays of *N*_*e*_ = 32–512 electrodes, which are a very small numbers compared to the full biological network size with *N* = *O*(10^6^–10^9^) neurons. This situation *N*_*e*_ << *N* has been called *subsampling*^51^,^52^,^53^.

The paradox occurs because models that present good power laws for avalanches measured over the total number of neurons *N*, under subsampling present only exponential tails or log-normal behaviors^53^. No model, to the best of our knowledge, has solved this paradox^10^. Our dynamic gains, which produce supercritical states like Γ^*^ = 1.01Γ_C_, could be a solution to the paradox if the supercriticality in the whole network, described by a power law with a supercritical bump for large avalanches, turns out to be described by an apparent pure power law under subsampling. This possibility will be fully explored in another paper.

### Directions for future research

Future research could investigate other network topologies and firing functions, heterogeneous networks, the effect of inhibitory neurons^30^,^42^, and network learning. The study of self-organized supercriticality (and subsampling) with GL neurons and dynamic neuron gains is particularly promising.

## Methods

### Numerical Calculations

All numerical calculations are done by using MATLAB software. **Simulation procedures:** Simulation codes are made in Fortran90 and C++11. The avalanche statistics were obtained by simulating the evolution of finite networks of *N* neurons, with uniform synaptic strengths *W*_*ij*_ = *W* (*W*_*ii*_ = 0), Φ(*V*) monomial linear (*r* = 1) and critical parameter values *W*_C_ = 1 and Γ_C_ = 1. Each avalanche was started with all neuron potentials *V*_*i*_[0] = *V*_R_ = 0 and forcing the firing of a single random neuron *i* by setting *X*_*i*_[0] = 1.

In contrast to standard integrate-and fire^12^,^13^ or automata networks^4^,^15^,^17^, stochastic networks can fire even after intervals with no firing (*ρ*[*t*] = 0) because membrane voltages *V*[*t*] are not necessarily zero and Φ(*V*) can produce new delayed firings. So, our criteria to define avalanches is slightly different from previous literature: the network was simulated according to equation (1) until all potentials had decayed to such low values that 
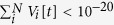
, so further spontaneous firing would not be expected to occur for thousands of steps, which defines a *stop time*. Then, the total number of firings *S* is counted from the first firing up to this stop time.

The correct finite-size scaling for avalanche duration is obtained by defining the duration as *D* = *D*_*bare*_ + 5 time steps, where *D*_*bare*_ is the measured duration in the simulation. These extra five time steps probably arise from the new definition of avalanche used for these stochastic neurons.

## Additional Information

**How to cite this article**: Brochini, L. *et al*. Phase transitions and self-organized criticality in networks of stochastic spiking neurons. *Sci. Rep*. **6**, 35831; doi: 10.1038/srep35831 (2016).

**Publisher’s note**: Springer Nature remains neutral with regard to jurisdictional claims in published maps and institutional affiliations.
